# New Frontier in Cancer Immunotherapy: Sexual Dimorphism of Immune Response

**DOI:** 10.3390/metabo15110686

**Published:** 2025-10-23

**Authors:** Nadeem Bilani, Nicole Charbel, Joe Rizkallah, Sam Sater, Firas Kreidieh

**Affiliations:** 1 Division of Hematology and Oncology, Department of Internal Medicine, Northwestern University, Evanston, IL 60208, USA; nadeem.bilani@nm.org; 2 Division of Hematology-Oncology, Department of Internal Medicine, American University of Beirut, Beirut P.O. Box 11-0236, Lebanon; nc47@aub.edu.lb; 3 Department of Diagnostic Radiology, American University of Beirut, Beirut P.O. Box 11-0236, Lebanon; jr56@aub.edu.lb; 4 Medical Oncology, HAS CONSULTING, Miami, FL 34984, USA

**Keywords:** sexual dimorphism, cancer immunotherapy, metabolomics, immune checkpoint inhibitors, sex hormones, tumor microenvironment, immunometabolism, metabolic biomarkers, precision medicine, sex-based differences

## Abstract

Sexual dimorphism influences immune responses, cancer progression, and therapeutic outcomes, yet its metabolic underpinnings remain underexplored. Metabolomics enables the comprehensive profiling of biochemical pathways that shape sex-based differences in immune function and immunotherapy efficacy. Meta-analytic data indicate that men achieve a larger overall survival benefit from immune checkpoint inhibitors than women (pooled hazard ratio 0.72, 95% CI 0.65–0.79 vs. 0.86, 95% CI 0.79–0.93), while women may experience higher major pathologic response rates in neoadjuvant settings. At the biomarker level, elevated kynurenine-to-tryptophan ratios—reflecting indoleamine 2,3-dioxygenase activity—and distinct lipidomic signatures associate with reduced immunotherapy efficacy and may vary by sex. Sex-specific differences in microbiome-derived metabolites, including short-chain fatty acids, further modulate systemic immunity and treatment response. Ongoing clinical investigations combine hormone modulation with immune checkpoint blockade and increasingly integrate metabolomic profiling to identify predictors of benefit and toxicity. This review will synthesize meta-analytic and mechanistic evidence on sex differences in immunotherapy outcomes, highlight metabolomic biomarkers linked to response, and summarize ongoing clinical trials that incorporate metabolomics to guide sex-aware precision oncology. Understanding sex-specific metabolic pathways can refine patient stratification and optimize immunotherapeutic strategies.

## 1. Introduction

Over the past two decades, strong evidence has emerged indicating the pivotal role of inflammatory immune cells in tumorigenesis [1]. While the immune system is responsible for detecting and eliminating cancer cells, immune dysregulation has been recognized as a key hallmark of cancer, with inflammation being associated with at least 25% of malignancies [2]. Cancer-associated inflammation contributes to genomic instability, epigenetic modifications, cancer cell proliferation, angiogenesis, and eventually, cancer dissemination. With increasing knowledge of the role of the immune system in cancer control, the advent of immune checkpoint inhibitors (ICIs)—which inhibit proteins that suppress T-cell activation, thereby reversing tumor-induced immune tolerance—has led to markedly improved cancer survival rates. Although tumor-infiltrating lymphocytes are integral to the efficacy of ICIs, the response to immunotherapy can be limited by insufficient antitumor immunity in the tumor microenvironment [2]. Innate and adaptive immune responses differ between female and male tumor microenvironments—a crucial factor in the immunotherapy response, known as sexual dimorphism [3]. Sexual dimorphism in the immune response is closely related to immune system homeostasis, primarily through differences driven by biologic sex hormones [3]. The male hormone testosterone is converted to its more biologically active form, dihydrotestosterone, by the enzyme 5α-reductase, and to the female hormone estradiol by the enzyme aromatase. The androgen receptor, located on the X chromosome, is expressed across various tissue types, including but not limited to adipose, muscle, bone, prostate, and other tissue types within the reproductive, cardiovascular, immune, neural, and haemopoietic systems [4]. Signal transduction via the androgen receptor involves receptor dimerization, ligand binding, interaction with cofactors, and eventually, DNA binding [5]. Notably, testosterone supplementation has been explored as a potential treatment for fatigue and cachexia in certain patients with cancer [6,7,8]. While clinical trials remain scarce, emerging evidence suggests that testosterone supplementation may also incur benefits on activity levels, physical performance [9] and mood [10]. However, androgens have also been associated with immunosuppressive effects, potentially interfering with immunotherapeutic efficacy [11].

Females typically mount stronger immune responses than males, primarily due to hormonal variations and differences in sex chromosome gene expression [12]. Consequently, women experience fewer infections than men but are more prone to autoimmune diseases [11]. Additionally, they tend to develop higher titers of protective antibodies following vaccination, though with a greater likelihood of adverse reactions [13]. On the other hand, male tumors are often more antigenic than female tumors, with a higher tumor mutational burden. However, tumor mutational burden is a less reliable predictor of immunotherapy response in men than in women. As a result, therapeutic approaches may need to be sex-specific: enhancing the immune environment in male patients and increasing tumor antigenicity in female patients [12]. These disparities affect immunotherapy efficacy, biomarker reliability, and potential treatment strategies.

The binding of sex hormones to their receptors has been shown to modulate immune function via both DNA binding-dependent and independent mechanisms [5]. As such, sex hormonal balance likely contributes to tumorigenesis through direct and indirect mechanisms. While tumor characteristics, such as programmed death-ligand 1 (PD-L1) expression, tumor mutational burden, and tumor-infiltrating lymphocyte status, are well-established predictive biomarkers for immunotherapy response, the role of sexual dimorphism is yet to be further described. Metabolomics provides a comprehensive assessment of biochemical pathways and could offer valuable information about sex-based differences in immune function and cancer immunotherapy efficacy. Sexual dimorphism extends to metabolic pathways, influencing nutrient utilization, energy production, and the accumulation of specific metabolites that can directly impact immune cell differentiation, activation, and effector functions [14,15]. Understanding these sex-specific metabolic alterations is crucial for deciphering the observed differences in cancer progression and treatment responses between males and females.

Beyond endogenous sex hormone activity, pharmacologic modulation also impacts immune and inflammatory responses. In males, finasteride—a 5α-reductase inhibitor that reduces dihydrotestosterone levels—has been associated with changes in immune regulation, including reductions in oxidative stress and the suppression of pro-inflammatory mediators such as cyclooxygenase-2, inducible nitric oxide synthase, and interleukin-8, thereby attenuating inflammatory signaling [16]. In females, hormone replacement therapy alters systemic estrogen and progesterone exposure, enhancing B-cell survival and antibody secretion and promoting T-helper 2–driven T-cell responses; however, these immune-stimulatory effects are also associated with an increased risk of autoimmune activation and disease flares [17]. These clinical examples illustrate how both physiologic and therapeutic hormonal changes contribute to sexual dimorphism in immune function and cancer susceptibility.

Studies suggest that ICIs tend to be more effective in male cancer patients, whereas ICIs combined with chemotherapy show better results in females [12]. The intricate interplay between metabolism and immune cell function, termed immunometabolism, is increasingly recognized as a crucial determinant of immune responses against cancer. Immunometabolism offers promising avenues for enhancing antitumor immunity by targeting metabolic pathways [18]. The interaction between tumor metabolites and T-cell dysfunction is critical in understanding and improving immunotherapy responses [19]. Targeting metabolic circuits that impede antitumor immunity and repurposing drugs that influence cancer metabolism may synergistically enhance immunotherapy effectiveness by reprogramming the tumor microenvironment [20].

In this narrative review, we summarize the existing literature exploring the interplay between sex chromosomes, sex hormones, and the immune system, emphasizing how sexual dimorphism may affect immunotherapy outcomes. A deeper understanding of sex-specific metabolic pathways may optimize immunotherapeutic strategies for cancer treatment and improve patient stratification and outcomes. This review will delve into how sex hormones shape distinct metabolic profiles within immune cells and the tumor microenvironment, leading to unique metabolic signatures that correlate with susceptibility to cancer and responsiveness to immunotherapy. We will specifically highlight recently identified metabolomic biomarkers that hold promise for predicting and monitoring response to ICIs and explore the integration of metabolomics into ongoing clinical trials to pave the way for sex-specific precision medicine in oncology.

## 2. Materials and Methods

We began this narrative review with a literature search through PubMed, Scopus, and Cochrane databases to capture peer-reviewed research articles—including reviews and meta-analyses—published on this topic between 2003 and 2025.

The search strategy involved combining the following terms with the Boolean operator “AND”: “Sex hormones”, “Immunotherapy”, and “Cancer”. Additionally, for metabolomics-specific content, terms like “metabolomics,” “metabolic biomarkers,” “immunometabolism,” “sex differences,” and “precision medicine” were used in combination with “cancer” and “immunotherapy.” All collected abstracts were surveyed by two independent researchers to confirm article relevance to the proposed study topic. Data extraction was performed on the included articles that met the review objectives.

Articles in any language were included provided that an English translation existed. These aforementioned search terms were also used to identify ongoing clinical trials on ClinicalTrials.gov to also survey unpublished studies on this topic.

## 3. Results

A total of 227 items were produced by the aforementioned search strategy. After screening for research published or initiated with regards to ongoing clinical trials, and filtering through a manual review of study titles and abstracts, this was pared down to 68 studies included in this narrative review. This included population-based studies commenting on sex-based disparities in patients treated with immunotherapy, pre-clinical or clinical studies identifying biologic mechanisms outlining the interplay between immunity and cancer outcomes, and clinical trials combining immunotherapy with either hormone supplementation or deprivation.

## 4. Discussion

### 4.1. Epidemiologic Data

Sex-based differences are evident in cancer incidence and mortality rates across ages and tumor types. Most malignancies with a clear sex difference impact men more than women, with male-predominant malignancies including bladder, colon, skin, liver, brain, and hematologic cancers [21,22]. These sex-based differences have been implicated in response to different treatment regimens, patterns of metastasis, and the expression of prognostic and predictive biomarkers [23,24]. Yet, sex is not consistently considered as a variable in the design of clinical trials. Specifically, women remain underrepresented in immunotherapy-related clinical trials [25].

A systematic review and meta-analysis published by Conforti et al. (2018) [26] included 20 randomized controlled trials of ICIs used predominantly in the context of advanced or metastatic melanoma and non-small-cell lung cancer. This study suggested that the magnitude of benefit on overall survival was sex-dependent, with a pooled hazard ratio of 0.72 (95% confidence interval [CI]: 0.65–0.79) in males treated with ICIs compared to men treated in control groups, compared to a pooled hazard ratio of 0.86 (95% CI: 0.79–0.93) in women [26]. Later in 2021, Conforti et al. [27] also assessed sex-based heterogeneity specifically in survival after immune checkpoint inhibitors targeting the PD-1/PD-L1 pathway for non-small-cell lung cancer expressing high PD-L1 levels in another systematic review and meta-analysis that included 1672 patients. The pooled hazard ratio comparing anti-PD-1/anti-PD-L1 therapy versus chemotherapy was 0.59 (95% CI: 0.50–0.69) for men and only 0.84 (95% CI: 0.64–1.10) for women. The data highlighted a concern that anti-PD-1/anti-PD-L1 monotherapy was highly effective in men but not in women, even in non-small-cell lung cancer expressing high PD-L1 levels [27]. Limitations of these data include the likelihood that confounders of overall survival—such as comorbid conditions including autoimmunity or immunodeficiency, lifestyle habits, subsequent-line treatments, medication interactions, or long-term treatment toxicity—were not captured in the meta-analysis, potentially influencing the observed greater effect size of benefit in men. On the other hand, another analysis by Graham et al. (2018) showed no significant difference in the advantages of nivolumab between men and women with metastatic renal cell carcinoma [28]. More recent meta-analyses continue to explore these differences. For instance, a systematic review and meta-analysis by Lai et al. (2024) [29] highlighted the importance of considering sex, line of therapy, cancer subtype, and PD-L1 status when assessing the risk versus benefit of ICIs. In the first-line setting, the pooled interaction hazard ratio (HR) for overall survival between men and women was 0.97 (95% CI: 0.91–1.04), indicating no significant difference. However, in subsequent-line therapy, the pooled interaction HR was 0.85 (95% CI: 0.77–0.95), suggesting a greater benefit for men compared to women [29]. Sexual dimorphism in response to immunotherapy may also vary by cancer type or be agent specific.

Survival may not fully convey efficacy outcomes, including the response rate (captured by pathologic response rates) and duration of response (captured by progression-free or disease-free survival). Another meta-analysis by Suay et al. (2023) observed that major pathological responses in neoadjuvant immunotherapy for non-small-cell lung cancer were significantly higher in females than in males [30]. Wang et al. (2019) found that both PD-1 and PD-L1 inhibitors alone or in combination with chemotherapy improved progression-free survival in male patients (HR of 0.67, 95% CI: 0.58–0.77), with non-small-cell lung cancer; however, in female patients, monotherapy with PD-1 inhibitors did not improve progression-free survival (0.95, 95% CI: 0.77–1.18) [31]. Finally, different outcomes in survival may be related to the varying side effect profiles of ICIs, with the risk of endocrinopathies being higher in women and the risk of neuromuscular immune complications appearing more common in men [32].

A meta-analysis published by Wallis et al. (2019) attempted to address these questions, and limitations from the initial review by Conforti et al. (2018) were addressed through greater inclusion of pertinent clinical trials and subgroup analyses by disease site, line of therapy, and class of immunotherapy. This updated stratified analysis including 23 randomized controlled trials demonstrated no statistically significant difference in the efficacy of immunotherapy in improving overall survival for the treatment of advanced cancers between men and women (I^2^ = 38%; *p* = 0.60) [26,33]. This meta-analysis included trials in the context of advanced-stage disease.

These analyses are limited by the exclusion of trials without sex-specific subgroup data. Further research is needed to further elucidate sex-based differences in response to immunotherapy by selected agent, disease stage, cancer type, and host biology.

### 4.2. Mechanistic Studies

**Figure 1:**
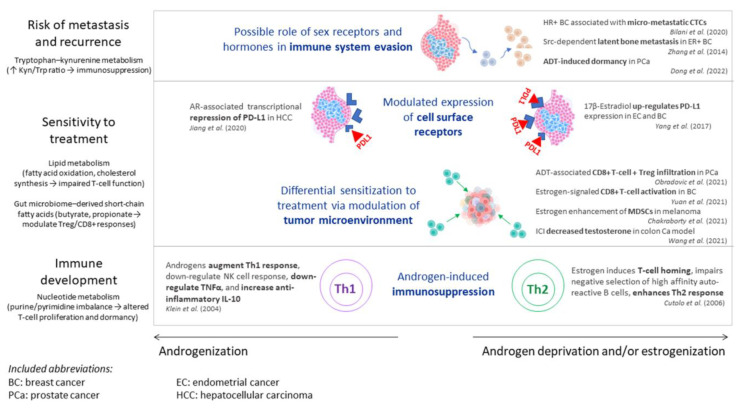
Mechanisms of interplay between immunity, sex hormones, metabolomic pathways, and cancer development, progression, treatment sensitivity, and recurrence. This schematic summarizes how sex hormones influence immune development, treatment sensitivity, and risk of metastasis or recurrence, with metabolomic pathways integrated alongside hormonal and immune interactions. Risk of metastasis and recurrence is influenced by hormone receptor signaling and immune evasion, in parallel with tryptophan–kynurenine metabolism (elevated Kyn/Trp ratio leading to immunosuppression). Sensitivity to treatment is modulated by hormone-driven expression of tumor cell surface receptors and changes in the tumor microenvironment, intersecting with lipid metabolism (fatty acid oxidation and cholesterol synthesis) and gut microbiome-derived short-chain fatty acids (butyrate and propionate). Immune development is shaped by androgens (promoting Th1 responses but inducing immunosuppression) and estrogens (enhancing Th2 responses and T-cell homing), with nucleotide metabolism influencing T-cell proliferation and dormancy. Together, these pathways illustrate the interconnected roles of sex hormones and metabolomics in shaping immune responses, tumor biology, and immunotherapy outcomes. Refs. [3,35,42,43,44,45,46,47,48,49,50]. Abbreviations: ADT, androgen deprivation therapy; AR, androgen receptor; BC, breast cancer; Ca, cancer; EC, endometrial cancer; ER, estrogen receptor; HCC, hepatocellular carcinoma; HR, hormone receptor; ICI, immune checkpoint inhibitor; IL, interleukin; Kyn, kynurenine; MDSC, myeloid-derived suppressor cell; NK, natural killer; PCa, prostate cancer; PD-L1, programmed death-ligand 1; Th1, type 1 helper T cell; Th2, type 2 helper T cell; TNF, tumor necrosis factor; Treg, regulatory T cell; Trp; tryptophan.

As depicted in Figure 1, multiple mechanisms through which sex chromosomes or sex hormones modulate the interplay between the immune system, cancer development, progression, response to treatment, and risk of recurrence have been described to date. This has been investigated in patients with, or models for, a diverse range of neoplasms, including breast cancer, prostate cancer, hepatocellular carcinoma, colon adenocarcinoma, and melanoma.

#### 4.2.1. Immune Development

The sex bias in immunity can be partially explained by gene diversity and dosage. The hormonal and immune abnormalities associated with inherited sex chromosome disorders such as Klinefelter with XXY in males and Turner syndrome with XO in females supports our understanding of X chromosome involvement in sex-biased immunity. Many genes on the X chromosome are associated with immune regulation, including IL-2R γ chain, IL-3R α chain, IL-13 α chain, IL-1R associated kinase 1 (IRAK1) TLR7, GATA1, FOXP3, and CD40L [34]. Men carry one X chromosome while women carry two, one of which is randomly transcriptionally inactivated. Throughout development, immune system differentiation—including innate and adaptive immunity—is further developed by sex hormones, including androgens and estrogens. Estrogen has been shown to enhance the immune system, compared to testosterone, which has a suppressive effect. Estrogen impairs the negative selection of high-affinity auto-reactive B-cells and leads to a Th2 response [35,36] characterized by robust humoral immunity through the activation of B-cells producing antibodies. Studies have shown that low testosterone levels are associated with higher B-cell counts [37] and poorer antibody responses to vaccination in men [38]. Androgens, on the other hand, while augmenting the Th1 response characterized by cell-mediated immunity via CD8 T-cells and macrophages, downregulate the natural killer response, decrease the proinflammatory cytokine tumor necrosis factor, and increase the production of anti-inflammatory interleukin-10 [39,40]. Testosterone has also been shown to limit the number of T-cells residing in the periphery [41]. This tempered immune response, as is the case in individuals with primary or secondary immunodeficiency disorders, is hypothesized to increase the risk of tumorigenesis due to poor or dysfunctional immune surveillance for neoplastic cells.

#### 4.2.2. Sensitivity to Treatment

Sex hormones may affect the sensitivity of cancer to treatment through multiple mechanisms. This may occur through modulated expression of tumor cell surface receptors affecting the recognition of tumors via antigenicity. Jiang et al. (2020) found that the androgen receptor was implicated in transcriptional repression of PD-L1 in animal models of hepatocellular carcinoma [42], whereas Yang et al. (2017) found that estradiol was associated with upregulated expression of PD-L1 [43].

ICIs such as atezolizumab, avelumab, and durvalumab block PD-L1, thus preventing neoplastic cells from suppressing the host immune response. However, sex hormones may also affect the efficacy of immunotherapy through changes to the tumor microenvironment. In a randomized study, Obradovic et al. (2020) found that androgen deprivation therapy was associated with improved CD8+ T-cell infiltration within the prostate tumor microenvironment through increased expression of chemokines (including CXCL10 and IL7) and antigen presentation machinery (MHC class I and II) by tumor cells [44]. Similarly, estrogen signaling has been linked to improved CD8+ T-cell activation in breast cancer mouse models [45]. Data from pre-clinical studies do not consistently indicate that androgen deprivation or estrogenization is associated with a more robust immune response. Chakraborty et al. (2021) found that estrogen signaling skewed the tumor environment in models of melanoma towards an immunosuppressive state via the recruitment of myeloid-derived suppressor cells, and that treatment with a selective estrogen receptor down-regulator increased the antitumor efficacy of the immune checkpoint blockade [46].

Finally, Wang et al. (2021) demonstrated cross-talk or dynamic interplay between immunotherapy and sex hormone levels, with anti-PD-L1 treatment decreasing serum levels of both testosterone and estradiol in male but not female mice models of colon cancer, likely through the disturbance of lipid metabolism and inflammation in the testes of male mice [47]. Castration of male mouse models was associated with enhanced antitumor efficacy of the immune checkpoint blockade. However, testosterone levels in tumor-bearing female mice treated with ICI therapy were, in fact, higher than levels in untreated mice, and lower than levels in tumor-free mice. This suggests a concentration–effect relationship that may be tumor type- and host sex-specific.

#### 4.2.3. Risk of Metastasis and Recurrence

Ongoing research continues to contribute to our understanding of tumor dormancy. Several studies have indicated that sex hormones may be implicated in the propensity for neoplasms to exhibit micro-metastasis and evade immune surveillance, leading to tumor recurrence. Positive hormone receptor status has been associated with the presence of circulating tumor cells in an analysis of a large retrospective registry for breast cancer in the United States [48]. Zhang et al. (2009) indicated that latent recurrence associated with estrogen-positive breast cancers may be associated with c-Src-mediated metastasis of neoplastic breast cancer cells to the bone, where latency is induced and immune evasion can occur [49]. Dong et al. (2022) used transcriptome profiling analyses to identify subtypes of prostate cancer more likely to become dormant after treatment with androgen deprivation therapy [50]. This has potential to not only lead to the development of a novel predictive gene signature for risk stratification of patients, but also an opportunity to identify pathologic subtypes where the role of sex hormones or receptors may play a more predominant role in immune evasion.

### 4.3. Metabolomic Biomarkers in Sex-Specific Immunotherapy Response

Metabolomics, the large-scale study of small molecules (metabolites) within biological systems, offers a powerful lens to capture the dynamic metabolic state of an individual and their tumor, providing insights into disease progression and treatment response. In the context of cancer immunotherapy, metabolic reprogramming within tumor cells and immune cells significantly influences the efficacy of treatments such as ICIs. Emerging evidence suggests that sex-specific metabolic profiles contribute to the observed dimorphism in ICI outcomes [14,15].

Several studies have begun to identify specific metabolomic biomarkers linked to ICI response, with some demonstrating sex-dependent associations. For instance, alterations in amino acid metabolism, particularly the tryptophan and arginine pathways, have been implicated in immune evasion and resistance to immunotherapy. Higher kynurenine-to-tryptophan ratios, indicative of increased indoleamine 2,3-dioxygenase activity, are associated with immunosuppression and may predict poorer ICI response [19]. While not always explicitly stratified by sex, future research should explore whether the prognostic value of such markers differs between male and female patients, given the sex differences in inflammatory responses and indoleamine 2,3-dioxygenase expression.

Lipid metabolism is another area of active investigation. Changes in fatty acid oxidation or cholesterol synthesis within T-cells can impair their anti-tumor function. Recent studies have highlighted sex-specific differences in lipid profiles in various cancers, which could influence immune cell function and ICI sensitivity. For example, Yu and Aboud (2024) highlighted the significant role of a disrupted lipidomic signature in glioblastoma pathology and therapeutic resistance, emphasizing the need to further elucidate how specific lipid metabolites and pathways contribute to tumor growth and proliferation [51]. Growing evidence of sex-specific lipid metabolism suggests that targeting these pathways may improve immunotherapy sensitivity. Furthermore, studies have explored how microglial immunometabolism endophenotypes are implicated in sex differences in Alzheimer’s disease [52], raising the possibility that similar sex-specific metabolic pathways could be relevant for therapeutic approaches in cancer.

The gut microbiome, which significantly impacts host metabolism and immune responses, has been shown to exhibit sexual dimorphism [53,54]. Metabolites produced by gut microbes, such as short-chain fatty acids, can modulate systemic immunity and influence ICI efficacy [55,56]. The gut microbiome exhibits sex-specific differences in composition and function, which can impact inflammatory responses and disease susceptibility. Future metabolomic studies should consider the interplay between sex, gut microbiome-derived metabolites, and immunotherapy outcomes.

Nucleotide metabolism also plays a crucial role in immune cell proliferation and function. The dysregulation of purine and pyrimidine pathways can affect T-cell activation and differentiation, potentially influencing the ICI response. Investigating sex-specific differences in these metabolic pathways could reveal novel biomarkers or therapeutic targets. Advances in understanding tumor-associated macrophage immunometabolism also offer therapeutic perspectives, emphasizing how metabolic adaptations influence macrophage function within the tumor microenvironment, which could have sex-specific implications [57,58].

Beyond biological insights, methodological aspects of metabolomic studies are crucial for interpreting sex-specific findings. Most cited investigations employed high-resolution platforms such as liquid chromatography-mass spectrometry (LC-MS) or gas chromatography-mass spectrometry (GC-MS), enabling broad coverage of lipid and amino acid pathways. Nuclear magnetic resonance (NMR) spectroscopy has also been applied for its reproducibility in quantifying circulating metabolites. Analytical pipelines typically distinguish between untargeted approaches, which generate global metabolite profiles for hypothesis generation, and targeted approaches, which quantify predefined metabolites of interest. Data processing frequently involves multivariate analyses such as principal component analysis (PCA) and partial least squares discriminant analysis (PLS-DA) to separate male and female metabolic profiles, followed by pathway enrichment analyses to link metabolite clusters with immune response pathways. More recent studies have also incorporated integrative multi-omics approaches that combine metabolomics with transcriptomics and proteomics to refine predictive biomarkers of immunotherapy response in a sex-specific context.

A summary of key metabolomic biomarkers, their pathways, and reported sex-specific associations is provided in Table 1.

### 4.4. Ongoing Clinical Trials

Supported by a growing body of retrospective studies and pre-clinical data, numerous randomized controlled trials were designed to explore the efficacy of combining immunotherapy with either hormone supplementation or deprivation. These are summarized in Table 2. Breakdown by cancer type is as follows: 14 trials in prostate cancer, eight trials in breast cancer, and one trial in endometrial cancer.

Beyond trials exploring direct hormonal interventions, a growing number of clinical investigations are integrating metabolomic profiling to gain a deeper understanding of treatment response and resistance in a sex-specific manner. For instance, studies are utilizing advanced metabolomic profiling techniques to analyze patient samples (e.g., plasma, urine, tumor tissue) before and during cancer treatment to identify metabolic signatures predictive of response or adverse events. For example, Jalota et al. (2023) identified specific pretreatment host metabolites that correlate with the severity and time-to-onset of acute toxicities like cytokine release syndrome and immune effector cell-associated neurotoxicity syndrome in patients receiving chimeric antigen receptor T-cell therapy [59]. Similarly, Gómez de Cedrón et al. (2025) conducted a randomized clinical trial demonstrating that formulated bioactive phenolic diterpenes can exert immune-metabolic effects in cancer patients undergoing various treatments, with potential clinical benefits and implications for personalized nutrition strategies [60]. The integration of multi-omics data, including metabolomics, transcriptomics, and proteomics, is expected to provide a more holistic view of the sex-specific biological landscape influencing immunotherapy outcomes [61]. These efforts are crucial for moving towards a precision medicine approach, where metabolomic insights, coupled with an understanding of sexual dimorphism, can guide patient stratification and personalized treatment strategies.

Most of the ongoing trials in Table 2 assess efficacy endpoints such as overall survival, progression-free survival, and pathologic response, in addition to safety and toxicity. As many of these studies are still in progress or have not yet reported results, comprehensive outcome data remain unavailable. Future analyses will be needed to incorporate these findings once mature data are published.

## 5. Conclusions

Sexual dimorphism in the immune response is closely related to immune system homeostasis, particularly with differences in immunity related to biologic sex hormones. Sex-based differences are evident in cancer incidence and mortality rates across ages and tumor types. Estrogen has been shown to enhance the immune system, compared to testosterone, which has a generally immunosuppressive effect. Moreover, estrogen impairs the negative selection of high-affinity auto-reactive B-cells and leads to a Th2 response, characterized by robust humoral immunity through the activation of B-cells producing antibodies. Most meta-analyses are limited by bias created by the lack of inclusion of trials that did not publish sex-subgroup analyses. Future trials should integrate translational methodologies into their design to clarify the mechanisms by which sex hormones shape tumor antigenicity and promote immune evasion. Sex hormones may affect the sensitivity of cancer to treatment through multiple mechanisms, including the modulation of tumor cell surface receptor expression, changes in the tumor microenvironment, and enhancing the propensity to exhibit micro-metastasis and evade immune surveillance, thus leading to tumor recurrence. Ongoing retrospective, pre-clinical, and randomized trials are investigating combinations of immunotherapy with hormone modulation. In the future, the evaluation of hormonal balance modulation—through supplementation or deprivation—should continue to be extended to malignancies traditionally considered unrelated to hormonal influence, i.e., outside of prostate and breast cancer. Our narrative review introduces the sexual dimorphism of the immune response to cancer treatment as a new frontier in cancer immunotherapy. The growing field of metabolomics provides an unparalleled opportunity to unravel these underlying sex-specific metabolic differences, allowing for the identification of novel biomarkers and therapeutic targets. By integrating metabolomic profiling into clinical practice, we can move towards a truly personalized approach to cancer treatment, optimizing immunotherapeutic strategies based on an individual’s sex and unique metabolic signature.

## Figures and Tables

**Table 1:** Selected metabolomic biomarkers linked to cancer immunotherapy response and sexual dimorphism.

Biomarker	Metabolic Pathway	Relevance to ICI Response	Reported Sex-Specific Associations
Kynurenine-to-tryptophan ratio (Kyn/Trp)	Tryptophan metabolism (IDO activity)	Elevated ratio indicates immunosuppression and poorer ICI response	IDO expression and inflammatory responses show sex differences
Arginine	Arginine metabolism	Depletion impairs T-cell proliferation and effector function	Sex differences in arginase activity suggested
Lipidomic signatures (fatty acid oxidation, cholesterol synthesis)	Lipid metabolism	Dysregulated lipid metabolism linked to T-cell dysfunction and resistance	Distinct lipidomic profiles observed between men and women in multiple cancers
Short-chain fatty acids (butyrate, propionate)	Microbiome-derived metabolites	Modulate systemic immunity and affect ICI efficacy	Gut microbiome composition and SCFA production differ by sex
Purine and pyrimidine intermediates	Nucleotide metabolism	Influence T-cell activation, proliferation, and dormancy	Potential sex-specific effects remain underexplored

Abbreviations: ICI, immune checkpoint inhibitor; IDO, indoleamine 2,3-dioxygenase; Kyn, kynurenine; SCFA, short-chain fatty acid; Trp, tryptophan.

**Table 2:** Registered clinical trials combining hormone supplementation or deprivation with immunotherapies, grouped by cancer type.

Study ID	Phase	Intervention(s)	Sponsor	Status
Breast cancer				
NCT02221999	Phase II	Paclitaxel + cisplatin ± goserelin/leuprolide or letrozole	RenJi Hospital	Active
NCT03280563	Phase I/II	Tamoxifen/fulvestrant/exemestane + atezolizumab ± targeted therapies	Hoffmann-La Roche	Completed
NCT02971748	Phase II	Tamoxifen/aromatase inhibitor/LHRH agonist (physician’s choice) + pembrolizumab	MD Anderson Cancer Center	Active
NCT02648477	Phase II	Antiestrogen + pembrolizumab vs. doxorubicin + pembrolizumab	City of Hope	Completed
NCT02971761	Phase II	Pembrolizumab + enobosarm (selective AR modulator)	City of Hope	Completed
NCT02997995	Phase I	Exemestane + tremelimumab	UNICANCER	Completed
NCT02778685	Phase II	Letrozole + pembrolizumab + palbociclib	City of Hope	Active
NCT02990845	Phase I/II	Pembrolizumab + exemestane + leuprolide	National Taiwan University Hospital	Terminated
Endometrial cancer				
NCT04046185	Phase I	Toripalimab + progesterone	Shanghai First Maternity & Infant Hospital	Unknown
Prostate cancer				
NCT04934722	Phase III	Pembrolizumab + enzalutamide + ADT vs. placebo + enzalutamide + ADT	Merck Sharp & Dohme	Active
NCT04191096	Phase III	Pembrolizumab + enzalutamide + ADT vs. placebo + enzalutamide + ADT	Merck Sharp & Dohme	Active
NCT02312557	Phase II	Pembrolizumab + enzalutamide	OHSU Knight Cancer Institute	Active
NCT04631601	Phase I/II	Acapatamab + enzalutamide vs. acapatamab + abiraterone vs. acapatamab + AMG404 vs. AMG404	Amgen	Terminated
NCT01688492	Phase I/II	Abiraterone + prednisone + ipilimumab	Memorial Sloan Kettering	Active
NCT02020070	Phase II	Degarelix + ipilimumab	Memorial Sloan Kettering	Active
NCT00170157	Phase II	Ipilimumab + leuprolide/goserelin + flutamide/bicalutamide	Mayo Clinic	Completed
NCT03016312	Phase III	Enzalutamide ± atezolizumab	Hoffmann-La Roche	Completed
NCT02787005	Phase II	Enzalutamide ± pembrolizumab	Merck Sharp & Dohme	Completed
NCT01867333	Phase II	Enzalutamide ± PROSTVAC-F/V-TRICOM	National Cancer Institute	Completed
NCT01875250	Phase II	Enzalutamide ± PROSTVAC-F/V-TRICOM	National Cancer Institute	Completed
NCT01696877	Phase I/II	Degarelix ± cyclophosphamide + GVAX	Johns Hopkins	Completed
NCT03753243	Phase II	Pembrolizumab + enzalutamide	Mark Garzotto	Unknown
NCT04946370	Phase I/II	Pembrolizumab + enzalutamide/apalutamide ± ^225^Ac-J591	Weill Cornell	Recruiting

Abbreviations: ADT, androgen deprivation therapy; AR, androgen receptor; LHRH, luteinizing hormone-releasing hormone.

## Data Availability

No new data were created or analyzed in this study. Data sharing does not apply to this article.
